# Impact of Stilbenes as Epigenetic Modulators of Breast Cancer Risk and Associated Biomarkers

**DOI:** 10.3390/ijms221810033

**Published:** 2021-09-17

**Authors:** Sebanti Ganguly, Itika Arora, Trygve O. Tollefsbol

**Affiliations:** 1 Department of Biology, University of Alabama at Birmingham, Birmingham, AL 35294, USA; sebantig@uab.edu (S.G.); itiarora@uab.edu (I.A.); 2 Integrative Center for Aging Research, University of Alabama at Birmingham, Birmingham, AL 35294, USA; 3 O’Neal Comprehensive Cancer Center, University of Alabama at Birmingham, Birmingham, AL 35294, USA; 4 Nutrition Obesity Research Center, University of Alabama at Birmingham, Birmingham, AL 35294, USA; 5 Comprehensive Diabetes Center, University of Alabama at Birmingham, Birmingham, AL 35294, USA; 6 Cell Senescence Culture Facility, University of Alabama at Birmingham, Birmingham, AL 35294, USA

**Keywords:** stilbene, polyphenols, breast cancer, prevention, epigenetics, biomarkers, methylation, microRNAs, cell-signaling

## Abstract

With the recent advancement of genetic screening for testing susceptibility to mammary oncogenesis in women, the relevance of the gene−environment interaction has become progressively apparent in the context of aberrant gene expressions. Fetal exposure to external stressors, hormones, and nutrients, along with the inherited genome, impact its traits, including cancer susceptibility. Currently, there is increasing interest in the role of epigenetic biomarkers such as genomic methylation signatures, plasma microRNAs, and alterations in cell-signaling pathways in the diagnosis and primary prevention of breast cancer, as well as its prognosis. Polyphenols like natural stilbenes have been shown to be effective in chemoprevention by exerting cytotoxic effects that can stall cell proliferation. Besides possessing antioxidant properties against the DNA-damaging effects of reactive oxygen species, stilbenes have also been observed to modulate cell-signaling pathways. With the increasing trend of early-life screening for hereditary breast cancer risks, the potency of different phytochemicals in harnessing the epigenetic biomarkers of breast cancer risk demand more investigation. This review will explore means of exploiting the abilities of stilbenes in altering the underlying factors that influence breast cancer risk, as well as the appearance of associated biomarkers.

## 1. Introduction

According to the 2020 global cancer statistics performed by GLOBOCAN, an initiative of the World Health Organization, breast cancer is the most prevalent form of cancer in women, and contributed to 11.7% of the total global cancer burden that was estimated to be 19.3 million cases in the year 2020 [1,2]. North American countries, including the United States, saw a substantial increase in the total number of reported incidences in female breast cancer patients in the years around 2007 due to rising awareness with respect to annual screening. Simultaneously, a lower number of reports on mortality rates reflected asymptomatic women’s willingness to adopt primary preventive measures by addressing external factors such as lifestyle, diet, age of conceiving a full-term pregnancy, duration of breastfeeding, and nulliparity. There is little to no control of factors such as somatic age; race; age of menarche; menopause; density of breast tissue; acquired and germline mutations in the form of deletion or truncation of tumor suppressor genes (TSGs), including *BRCA1/2, TP53, STK11, CD1,* and *PTEN;* and upregulation of oncogenes such as *ERBB2, c-MYC,* and *PIK3C* [3,4,5,6]. However, there is still a degree of control that medical science possesses in reversing epimutations or epigenetic changes that silence or activate the aforementioned genes, which serve as biomarkers of breast cancer. The current research not only ensures maximum survival rates, but also focuses on preventing the poor quality of life that breast cancer malignancies entail through the prevention of the occurrence of disease pathologies in high-risk individuals through the employment of primary prevention.

Primary prevention includes lifestyle changes; risk-reducing mastectomy; and undergoing simultaneous treatment with selective estrogen receptor modulators (SERMs) such as tamoxifen, raloxifene, lasofoxifene, and aromatase inhibitors (AI). Nelson et al., in 2019, in a very extensive meta-analysis, addressed some of the important questions regarding the effectiveness of adopting such a precautionary intake of SERMs by healthy individuals belonging to breast cancer-risk groups in the successful prevention of disease-occurrence and mortality over the long term [7]. SERMs indeed reduce the risk of breast cancer susceptibility significantly in estrogen receptor-positive (ER+) breast cancers, but some limitations in the meta-analysis caused by variability in the duration of drug administration and disregard with respect to the ages of the subjects undergoing the clinical trial has left grounds for skepticism [7]. Their body of work acknowledges the fact that the intake of SERMs in asymptomatic women as a measure of primary prevention is a rarely practiced clinical procedure because of the health-related concerns of the subjects. These include the long-term and short-term side-effects of these drugs, including increased risk of endometrial hyperplasia and endometrial cancer, blood clotting, menstrual abnormalities, decreased bone and muscle density, hot-flashes, body pains, and sexual dysfunction, as well as other consequences. Additionally, tamoxifen and raloxifene are synthetic derivatives of stilbenes that may interfere with the normal functioning of off-target tissues, causing the death of healthy cells in vivo [3,8,9,10,11], leading to considerable interest in finding less harmful derivatives of plant polyphenols such as stilbenes, and optimizing their activity within the cell.

Epigenetics encompasses all the heritable, reversible changes in gene expression, without the genetic code becoming altered. A major epigenetic mechanism in mammals is the methylation of DNA at the CpG islands of the gene promoters, specifically at the fifth carbon of cytosine in the DNA backbone. Other important epigenetic mechanisms consist of methylation, phosphorylation, ubiquitination, SUMOylation, and acetylation of histone molecules and the influence of non-coding RNAs (ncRNAs), rendering a change in the conformation of the DNA. These covalent additions to the DNA structure modulate the accessibility of the DNA to different transcription factors, thereby upregulating or down-regulating a specific gene. The enzymes that are responsible for causing these epigenetic marks are called “writers”, which include DNA methyltransferase (DNMT), histone methyltransferase (HMT), and histone acetylase (HAT). The enzymes involved in removing epigenetic marks are called “erasers”, and include histone deacetylases (HDACs), DNA demethylases, and histone demethylases. The enzymes responsible for recognizing these marks and conducting downstream signaling are termed “readers” and are characterized by proteins with two types of domains—recognition and effector domains. Aberrant epigenetic marks can upregulate oncogenes or down-regulate tumor suppressor genes, and hence hold immense importance. The gene expression of the writer, reader, and eraser proteins, as well as their activity, can also be monitored to discover new biomarkers of breast cancer susceptibility.

## 2. Scope of Primary Prevention of Breast Cancer Using Phytochemicals Including Stilbenes

Phytochemicals exert their anti-tumorigenic effect by modulating the gene expression of writer and reader proteins, by changing the pharmacokinetics of the same proteins or by potentiating the traditional chemopreventative methods [12,13,14].

**Figure 1:**
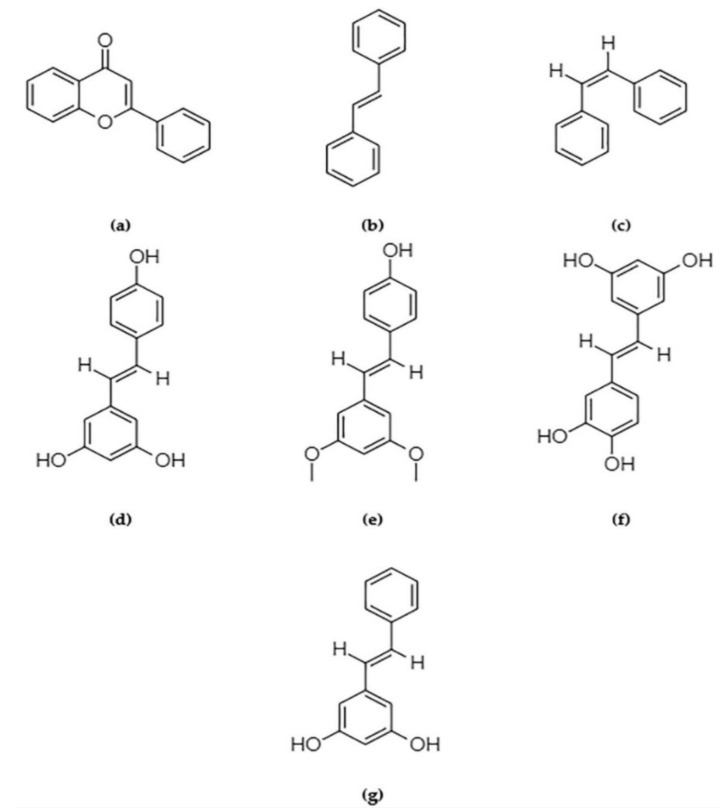
Structural difference between the molecular skeleton of flavones and stilbenes (**a**–**c**): (**a**) 15 carbon skeleton of flavonoids featuring a benzo-γ-pyrone structure; (**b**) trans-stilbene; (**c**) cis-stilbene; (**d**) resveratrol; (**e**) pterostilbene; (**f**) piceatannol; (**g**) pinosylvin. All of the chemical formulas are obtained from the PubChem database and are drawn with the help of ChemDraw.

Flavones and flavonoids (Figure 1a) represent the largest group of polyphenolic phytochemicals that have been abundantly studied. Currently, FDA-approved green tea catechins like epigallocatechin-3-gallate (EGCG), a naturally occurring flavone, is undergoing clinical trials (registered at clinicaltrials.gov as NCT00917735). The trial revealed no significant effect of EGCG in reducing breast cancer risk in postmenopausal women contrary to the dated observational studies [15]. Flavones such as apigenin [16] and luteolin [17], and isoflavones such as genistein and daidzein [18] have been evaluated in vitro and in vivo, and have shown promising results in the chemoprevention of breast cancer. Selvakumar et al. highlighted the mode of activity of flavonoids extensively [18]. EGCG increases the formation of S-adenosyl-L-homocysteine, which acts as a DNMT1 inhibitor and hence appears to be important for managing the methylated biomarkers discussed in the previous section. EGCG, when paired with an HDAC inhibitor like suberoylanilide hydroxamic acid (SAHA) or vorinostat, and administered to triple-negative breast cancer (TNBC) cell lines, has been shown to induce apoptosis and prevent metastatic tendencies by down-regulating the apoptosis inhibitor gene *cIAP2* [13,18,19]. This combination has also been found to repress the expression of microRNAs such as miR-221/222, which is attributed to the cell-renewal capabilities in TNBC cell lines, thus maintaining the PTEN/AKT/mTOR/NF-κB expression necessary for normal stem-cell maintenance [19,20]. Genistein, found in soy products, is a controversial phytochemical that can reduce cancer risk by increasing nitric oxide bioavailability, thereby increasing oxidative stress and DNA damage and stalling cell cycle progression. Genistein promotes apoptosis by targeting proteins like BCL-2, BAX, and caspase3 functions and modulating NF-κB, PI3KC/AKT, ERK1/2, and MAPK pathway downstream signaling. However, it may promote cancer progression by upregulating estrogen receptor signaling [21,22]. Evidence has shown that genistein is capable of rendering epigenetic marks such as acetyl-H3 and H3K4me3, which are conducive to TSG (p21 and p16) expression [23].

Lignans are phytochemicals that are obtained from sources like flaxseeds, that also show promise in reducing breast cancer risk, with minimal side effects [24]. Cruciferous vegetables such as broccoli, watercress, and cabbage harbor two of the most potent phytochemicals that prevent breast cancer—phenethyl isothiocyanate (PEITC) and sulforaphane. Sulforaphane down-regulates HDAC6 expression, subsequently elevating global histone acetylation. This triggers PTEN-mediated tumor suppression in the form of autophagy in TNBC cell lines [25,26,27]. Sulforaphane also enriches H3ac, H3K9ac, and H4ac marks, which confer euchromatinization and block H3K9me3 and H3K27me3, and promote heterochromatinization in the *hTERT* gene, thus enabling repressors to bind. The product of *hTERT* gene prevents telomere shortening and therefore prevents cell death, which attributes stemness in cancer cells. Sulforaphane is also capable of demethylating selective CpG sites of *hTERT,* resulting in its repression [28].

One naturally occurring phytochemical found in grape skin, which has the potential to epigenetically decrease breast cancer risk, is resveratrol (3,5,4′-trihydroxy-trans-stilbene; Figure 1d). This compound belongs to the polyphenolic group of stilbenes (Figure 1b,c). Stilbenes are naturally occurring compounds derived from plants like grapevine, sorghum, pine, spruce, and mulberry, and the kinds that contain a 1,2-diphenylethylene nucleus and are used by the plants for protecting themselves from external attacks of pests, microbes, and UV exposures [29]. It can be consumed as a part of the regular diet, with no apparent toxicity other than against breast cancer cells and progenitors, although it has not yet been approved by the FDA as a dietary supplement [30]. Sinha et al. reported the mechanisms involving the chemistry by which resveratrol epigenetically suppress proliferative signals of breast tumors and the subsequent risk reduction. Resveratrol is anti-methylation and pro-acetylation, given its capability to inhibit DNMT1, DNMT3A, DNMT3B, HDAC1, and MeCP2 [31]. Computational predictions have shown that resveratrol might also have significant interactions with epigenetic readers such as BRD4 bromodomain 1, which reads acetylated histone lysine residues and plays significant roles in cell proliferation repression in cancer [32,33]. Recent studies have emphasized a resveratrol-derivative referred to as pterostilbene (trans-3,5-dimethoxy-4-hydroxystilbene; Figure 1e), which has a higher bioavailability and potency compared with resveratrol for inhibiting the growth of cancer cells and cancer stem cells in cervical cancer [34]. Studies have shown that contrary to single stilbene treatment, a combination of resveratrol and pterostilbene modulates global DNA methylation by targeting DNMT and histone acetylations by inhibiting m (an HDAC), causing an enrichment of acetyl-H3, acetyl-H3K9, and acetyl-H4 active chromatin marks, thereby inhibiting cancer cell proliferation. This same combination is successful for converting ERα- breast cancer cells into ERα+ cells, thereby sensitizing the cells to chemopreventive drugs [12]. Pterostilbene, which is safe to consume, is therefore likely an important player in the primary prevention of breast cancer. Lesser-known stilbenes include piceatannol (trans-3,3′,4′,5-tetrahydroxystilbene; Figure 1f) and pinosylvin (3,5-dihydroxy-trans-stilbene; Figure 1g), which act as resveratrol analogs and possess a similar HDAC inhibitory function [29,35]. Because of their lower levels of side effects, stilbenes can be easily integrated into the diet of individuals who are prone to breast cancer tumorigenesis.

## 3. Scope of Primary Prevention in Breast Cancer from a Detailed Epigenetic Perspective

The five established molecular subtypes of breast cancer are luminal A, luminal B, ERBB2/HER2-overexpressing, basal-like breast cancer (BLBC), and normal-like tumors, based on the expression levels of estrogen receptor (ER), progesterone receptor (PR), Human Epidermal Growth Factor receptor 2 (HER2), and Ki-67. Ki-67 is considered as one of the significant biomarkers in women with atypical hyperplasia, which changes depending on time. Tsang et al. (2020) extended our current understanding on the classification of molecular subtypes of breast cancer by subdividing the BLBC further into CNA-quiet (copy number alterations quiet), 1q/16q, chromosome 8 associated, CNA-high (copy number alterations-high), and mixed subtypes [36,37]. Breast cancer can be histologically classified as ductal and lobular, but a single tumor often consists of cells from diverse molecular subtypes, making it a heterogeneous disease by nature. Despite significant overlap between the histological and molecular subtyping of breast cancer, the latter, when applied to construct a spatial map of the entire breast tumor architecture, assists clinicians with a better prognosis of the disease and facilitates their ability to formulate the most effective therapeutic approach tailored specifically to a given patient [38,39,40,41]. 

### 3.1. Genetic and Epigenetic Biomarker Landscape: Breast Cancer Risk Factors and Susceptibility

#### 3.1.1. DNA Methylation as Biomarkers

DNA methylation can be considered at two levels—focal or global. Focal DNA methylation deals with the methylation landscape on a single gene or locus basis, while global methylation accounts for the total 5-methylcytosine content in a biopsy sample methylome. There is a pattern in the focal counterpart; methylation at the upstream promoter of a gene is often associated with transcription repression, while methylation within the gene body is associated with an increased expression [42,43]. In HER2-enriched breast cancer patients or high-risk individuals, global methylation levels serve as successful biomarkers. Global methylation levels are lower in breast cancer patients and higher in normal individuals, especially at the repeat sequences like LINES or Alu, thereby keeping genomic instability in check [44,45,46]. However, there are some limitations to considering global methylation as a stable and strong biomarker for breast cancer risk determination. As pointed out by Ennour-Idrissi et al., the methylation signatures are subject to reversibility and variability based on tissue type [47]. To increase the precision of global methylation as a biomarker for breast cancer risk detection, collective methylation change in the global CpG landscape is being considered [48].

According to Knudson’s two hit hypothesis, for successful oncogenesis, both alleles of a tumor suppressor gene must be malfunctioning and silenced on one copy of a gene, and epigenetic alterations might provide a means for this process. From a gene-specific point of view, Nindrea et al. showed that hypermethylation at the *BRCA1* promoter can act as “hit” by down-regulating transcription and initiating loss-of-function, thus disrupting the DNA damage repair response [49,50], which serves as an excellent biomarker for elevated risk of hereditary triple-negative breast cancer (TNBC) cases [51]. Gene body hypermethylation of *ATM*, a breast cancer susceptibility gene that codes for downstream signaling proteins for cell cycle arrest, can lead to the early onset of breast cancer in women, and thus is a very useful biomarker [52]. Promoter hypomethylation in *PALB2*, a breast cancer susceptibility gene that localizes the *BRCA2* gene at the site of DNA damage, has been established as a biomarker for sporadic breast cancer [53]. Similarly, Masood et al. showed that hypermethylation in the 600 base pair region of the *hTERT* gene promoter can act as a potent biomarker for breast cancer diagnosis [54]. In the case of sporadic breast cancer, occasional rigorous screening of CpG landscapes of known breast cancer risk genes in healthy individuals may be the most logical way of assigning biomarkers. Ennour-Idrissi et al. provides information on seven significant genes that set breast cancer-prone individuals apart from resistant individuals [55]. There are also studies conducted on promoter methylation states of 100 genes, including *BRCA1, CCND2, BCL2, MDR1, IL10,* and *TWIST*, recorded to understand how alteration between hypermethylation and hypomethylation over time contributes to select breast cancer susceptibility biomarkers [56]. The list of breast cancer biomarkers involving DNA methylation continues to increase on a regular basis. 

#### 3.1.2. Histone Modifications as Biomarkers

Compared with DNA methylation, histone modifications are less constant epigenetic marks present on nucleosome structures that can change with every occasion of transcription. This helps to explain the scarcity of literature on anomalies in histone modifications as biomarkers for breast cancer risk assessment. Acetylation of histones is directly associated with euchromatic (open) and heterochromatic (tight) organization of DNA, which alters the accessibility to different transcription factors. Gene upregulation can often be found when acetyl groups are added to histone N terminal lysines (H3K9, H3K14, H3K18, H3K23, H4K5, H4K8, H4K12, and H4K16) [57]. Methylation at histone lysines (H3K9, H3K27, and H4K20, and histone arginine residues at H3R2me2a, H3R8me2a, H3R8me2s, and H4R3me2s) are associated with heterochromatin formation and gene-repression, while at H4R3me2a, H3R2me2s, H3R17me2a, and H3R26me2a, methylation confers an open chromatin structure [58,59].

There are other histone modifications such as ubiquitination, sumoylation, and phosphorylation. In patients who have already been diagnosed with breast cancer and are undergoing cancer surgery and treatment, global histone modifications have been shown to be an efficient tool for evaluating metastatic status, survival, and likelihood of relapse, as well as other outcomes [60,61].

#### 3.1.3. Non-Coding RNA as Biomarkers

Non-coding RNAs (ncRNAs) are short fragments of RNA transcribed from the non-coding regions or regions of DNA that may or may not be originated from ultraconserved elements [62]. ncRNAs may possess certain functions, such as regulating the interactions between different proteins with genomic particles, enhancing or repressing transcription rates, influencing alternate splicing, the ability to change 3D conformation of genomic DNA, and maintaining genomic stability, to mention a few [63]. The ncRNAs that span only a few (19–25) nucleotides are called microRNAs (miRNAs), and those above 200 nucleotides are called long non-coding RNAs (lncRNAs) [64]. They serve as important biomarkers for breast cancer risk determination and are non-invasively detectable from circulating blood as they are transported around the periphery of the body within the exosomes. Farina et al. discovered a panel of 2500 miRNAs related to breast cancer and found six with an abnormal presence in the blood in apparently healthy individuals that could serve as a biomarker for high-risk individuals with tendencies for future breast tumorigenesis [65,66]. Short nucleotide polymorphisms (SNPs) in lncRNAs are often hotspots of methylation and, like DNA methylation, these epigenetic marks can upregulate or down-regulate the activity of the lncRNAs [62,67]. Probing ncRNA activities is achievable, which makes tagging them as biomarkers easier [68]. Table 1 enlists such non-coding RNAs associated with breast cancer risk.

In addition to RNAs, there are also cell-free DNAs and proteins with methylation marks circulating in the bloodstream that can act as epigenetic biomarkers of cancer risk assessment [43,69].

**Table 1:** A non-exhaustive table of relevant ncRNAs associated with breast cancer risk prediction serving as biomarkers.

ncRNA	Therapeutic Significance	Breast Cancer Subtype	Reference
miR-21-3p, miR-21-5p, and miR-99a-5p, miR-1246, miR-1307-3p, miR-4634, miR-6861-5p, and miR-6875-5p	Risk prediction and early detection and overall survival	Unspecified	[43]
Panel of 8 miRNAs (miR-139-5p, miR-10b-5p, miR-486-5p, miR-455-3p, miR-107, miR-146b-5p, miR-324-5p, and miR-20a-5p)	Predicting risk of relapse	Triple-negative breast cancer	[70]
Panel of 6 miRNAs (miR-3124-5p, miR-1184, miR-4423-3p, miR-4529-3p, miR-7855-5p, and miR-4446-3p)	Breast cancer risk prediction	Unspecified	[65]
miRNA-191, miRNA-484, miR-16, miR-25, miR-222, and miR-324-3p	Breast cancer risk predictor	Unspecified	[71]
Panel of 4 miRNAs (hsa-miR-21, hsa-miR-494, hsa-miR-494, and hsa-miR-183)	Metastatic risk prediction	Unspecified	[72]
Panel of 4 miRNAs (miR-1246, miR-206, miR-24, and miR-373)	Early diagnosis of breast cancer	Unspecified	[66]
lncRNAs like *PVT1, MAPT-AS1, LINC00667*, and *LINC00938*	Predicting breast cancer survival	Unspecified	[73]
Panel of miR-127-3p, miR-148b, miR-376a, miR-376c, miR-409-3p, miR-652, and miR-801	Distinguishing healthy women from women carrying benign or malignant breast tumors with more accuracy in younger individuals	Unspecified	[74]
miR-200a, miR-200b, miR-200c, miR-210, miR-215 and miR-486-5p	Metastasis onset predictor	Unspecified	[74]
mRNA-lncRNA conjugate (mRNA species for *FCGR1A, RSAD2, CHRDL1,* and the lncRNA species for *HIF1A-AS2* and *AK124454*)	Predicting risk of relapse	Triple-negative breast cancer	[32]

## 4. In-Practice Clinical Methods for Addressing Primary Prevention

As previously mentioned, tamoxifen and raloxifene are FDA-approved commonly prescribed synthetic stilbene derivatives successful in chemoprevention if administered for five years, in both pre- and post-menopausal women, but might have adverse effects on other body tissues. There is another major drawback with tamoxifen chemotherapy in patients. ER+ cancer cells can develop resistance against tamoxifen due to an array of reasons, such as acquired perpetuation of repressive methylation marks in CpG islands of the estrogen-sensitive gene promoter [75,76,77,78,79,80,81]. Tamoxifen is found to be the most effective in individuals with a high risk of ER+ breast cancer, because it is a strong antagonistic competitor of estrogen and prevents estrogen-related growth signaling when it binds to the estrogen receptor in breast epithelia. Recent findings have indicating the potential evolution of superior derivatives of tamoxifen, such as endoxifen, which has a better affinity towards estrogen receptors, with better pharmacokinetics, allowing for greater bioavailability in a time-dependent manner [82]. This is currently undergoing a clinical trial, as indicated on the NIH US National Library of Medicine website clinicaltrials.gov.

In 2018, the FDA approved a poly (ADP-ribose) polymerase (PARP) inhibitor called talazoparib (TALZENNA, Pfizer Inc.) for patients who are carriers of the germline *BRCA* mutation and have HER2- metastatic breast cancer. Pulliam et al., in 2018, showed that a combination of talazoparib and DNMT inhibitor guadecitabine rendered promising results in modulating DNMT1 action, thereby altering the DNA damage repair response, increasing cellular concentration of caspase 3 and finally asserting cytotoxic effects on cancer stem cells with minimal side-effects in contrast with what has been observed with the traditional chemotherapy [83,84].

**Figure 2:**
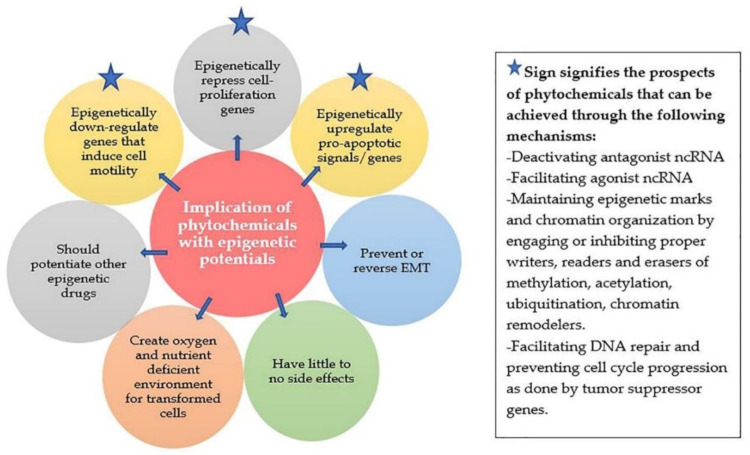
Factors that an effective phytochemical should control epigenetically for controlling cancer risk.

The basic methods to prevent breast cancer epigenetically are not significantly different from genetic approaches. A schema has been reported by Hanahan et al. summarizing feasible methods of managing breast cancer [85]. There is a plethora of research regarding primary prevention of breast cancer through epigenetic means, although not as many phytochemicals and their derivatives have been identified that can be adopted as a part of preventing the appearances of the first signs of breast cancer. Figure 2 demonstrates some basic qualities that phytochemicals should possess in order to be considered as efficient epigenetic chemopreventive compounds. The following paragraphs summarize some novel epigenetic prospects of stilbenes that have relevance for its consideration as a potent chemical to reduce the risk of breast cancer.

### 4.1. Role of Stilbenes in Differentially Modulating DNA Methylation of Genes and Gene Loci

Ongoing epigenetic research is mostly interested in discovering the mechanisms by which stilbenes differentially influence DNA methylation in both tumor suppressor genes and oncogenes or how they differentially methylate at two different CpG loci of the same gene. Harnessing these mechanisms can be the key to reversing the epigenetic marks that appear early at the onset of tumorigenesis; however, the biochemistry involved in this process is still elusive. In their experiment on both mild and aggressive breast cancer cell lines of MCF10A1a and MCF10A1h, Beetch et al. discovered 113 highly specific targets of resveratrol and pterostilbene. They also identified the *SALL3* gene (sal-like 3), which is upregulated by these two stilbenes, which in turn down-regulates DNMT3A, binding to the promoters of tumor suppressor genes like *SEMA3A* [86]. As a result, a hypomethylation state is created on the silenced tumor suppressor gene, which mimics the wild-type methylation state, restoring its expression. Furthermore, these stilbenes influence Nuclear Factor 1C protein, a tumor suppressor that localizes heavily on the *SEMA3A* promoter. Similar patterns of reactivating silenced tumor suppressor genes such as *p16, CCND2, APC*, and *RASSF1A* by inducing demethylation with trans-resveratrol have been reported by Zhu et al., supporting the idea that resveratrol can play important roles in primary chemoprevention [87]. However, there are drawbacks to this idea, because resveratrol has multiple off-target effects that also require further investigation.

The selective nature of resveratrol and other similar stilbene compounds were discussed by Aldawsari et al. through in silico molecular docking simulations, where they showed the similarity in the chemical structure between resveratrol and some recently identified small molecules, including methylenedisalisylic acid, which binds to DNMT3As and DNMT3Bs at the catalytic site or at the cofactor binding site (S-adenosylhomocysteine) with a high degree of specificity, and inhibits their activity by preventing DNA binding. They also conducted in vitro work that showed that resveratrol is more potent when converted into a hydroxylated hybrid salicylate derivative [88].

### 4.2. Role of Stilbenes in Differentially Modifying Histones

**Figure 3:**
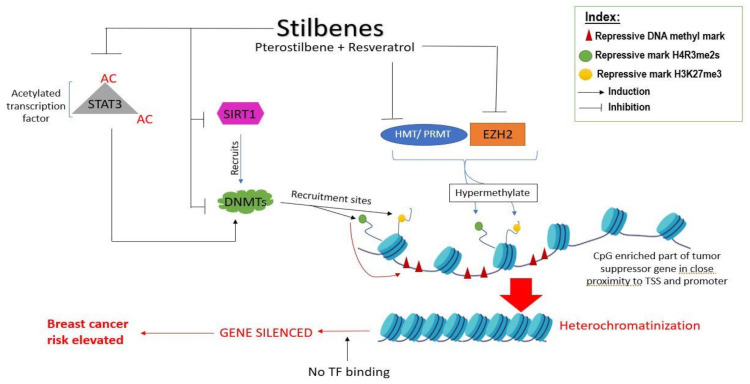
A schematic representation of the mechanisms involved in the suppression of TSGs in breast cancer and its reversal by stilbenes causing a reduction in breast-cancer risk.

**Figure 4:**
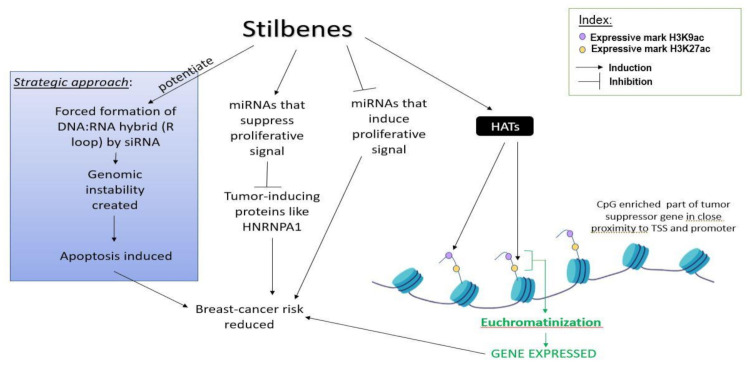
Schematic representation of the mechanisms involved in the expression of TSGs and the role of the stilbenes involved in this process.

It is a well-known fact that histone acetylation regulates DNA conformation, thereby regulating the access of transcription factors and enzymes like DNMTs to the DNA. A combinatorial dosage of resveratrol and pterostilbene has been shown to down-regulate SIRT1, an HDAC III responsible for leading DNMTs to hypermethylate promoters and silence TSGs (Figure 3) [14]. Chatterjee et al. showed that resveratrol can successfully down-regulate the oncogenic activity of PRMT5 (protein arginine methyltransferases) and EZH2 (catalytic domain of Polycomb repressive protein 2) by reducing silencing histone methyl marks H4R3me2s and H3K27me3 from TSGs (Figure 3). In contrast with this, they also showed that HATs are positively influenced by resveratrol, leading to the increase of expressive histone marks like H3K9ac and H3K27ac on the histones in proximity of the TSG (*BRCA1, p53,* and *p21*) promoters, as shown in Figure 4. Removal of the H3K27me3 mark also prevents DNMT1 and DNMT3A binding to the promoters of TSGs like *BRCA1*, leading to its expression and causing *p16*-dependent cell senescence to occur [89]. Not just histones, but transcription factors can directly become acetylated, which subsequently hypermethylate the promoter of tumor suppressor genes, leading to the gene suppression. For instance, as shown by Lee et al., acetylated transcription factor STAT3 can silence tumor suppressor genes in basal-like breast cancer tissues, which can be reverted by stilbenes like resveratrol and pterostilbene. As a downstream effect of resveratrol on inhibiting STAT3 acetylation, the *ERα* gene is demethylated and its expression is upregulated, which provides an opportunity to TNBC patients to undergo hormonal therapy [90,91]. Besides STAT3 acetylation, STAT3 phosphorylation also plays an important role in tumorigenesis, and stilbenes can successfully prevent this process [91,92]. A hybrid derivative of pterostilbene and vorinostat (hydroxamate) has been shown to bind to the SH2 domain of STAT3 with stable interactions at arginine and serine residues, preventing STAT3 from interacting with DNA. This conjugate has also been proven to inhibit HDACs [91].

### 4.3. Role of Stilbenes in Differentially Modulating the Activity of Non-Coding RNAs and Preventing Breast Cancer Initiation

Hagiwara et al. showed that both pterostilbene and resveratrol, in both their natural form and demethylated condition, are capable of upregulating Argonaute2 protein and thereby increased the expression of tumor-suppressive microRNAs like miR-16, miR-141, miR-143, and miR-200c in the triple negative breast cancer cell line MDA-MB-231 [93]. Otsuka et al.’s work on breast cancer-associated tumor-suppressive miRNAs (miR-34a, miR-424, and miR-503) demonstrated that these microRNAs are upregulated by resveratrol, which in turn suppresses tumor-inducing protein HNRNPA1 (heterogeneous nuclear ribonucleoprotein A1) [94]. A summary is depicted in Figure 4.

There are indirect ways by which stilbenes can modulate non-coding RNAs, in favor of reducing the risk of breast cancer. Stilbenes can influence the three-dimensional structure of R-loops formed by DNA:RNA triple strand hybrids that are known to regulate normal cellular functions such as transcription, DNA replication, and telomere maintenance via epigenetic control. This involves subsequent prevention or allowing the reader or writer proteins of methylation to interact with the gene regulatory regions. Loss-of-tumor-suppression function of BRCA2, impaired ATP-dependent chromatin remodeler protein SWI/SNF complex, and stress of reactive oxygen species can give rise to sporadic R-loops, which can act as biomarkers for genomic instability and are suitable targets for the DNA damage repair response. Thus, R-loops or similar DNA:RNA complex structures are hotspots for targeting chemopreventive measures in cancer patients. From a strictly strategic point of view, one of the ways that primary chemoprevention works in cell cultures is through delivering synthetic small interfering RNA (siRNA) to the DNA duplex of cancer cells or progenitors via the transfection of the micelle-bound RNA interference (RNAi) protein complex, thereby forming a complex DNA:RNA hybrid that resembles R-loop. Stilbenes and stilbenoids, when amalgamated to the end of siRNA of a RNAi complex, have been shown to increase the uptake of these micelle-bound RNAi complexes by the cancer cells, thereby increasing the effectiveness of RNAi technology [95,96]. Stilbenes might therefore be used to potentiate RNAi-based therapeutics and topical or intratumoral siRNA vaccine (for precision delivery). Individuals at high risk of breast cancer might benefit from this kind of therapeutics. While there could be drawbacks to this approach, further investigations and trials are required.

Epithelial-to-mesenchymal transformation is a normal cellular process needed for embryogenesis and wound-healing, which is characterized by loss of cell−cell and cell−matrix adhesion and the gain of motile features in epithelial cells, which is also a hallmark for cancer initiation and progression. Huang et al. showed that pterostilbene can prevent the epithelial-to-mesenchymal transformation (EMT) and promote mesenchymal-to-epithelial transformation (MET) by increasing the expression of lncRNA H19 [97]. LncRNA H19 acts as a differential sponge of microRNAs miR-200b/c and let-7b. This leads to modulating their targets guanosine triphosphatase-activating protein gene *Git2* and Cytohesin-3 protein coding gene *Cyth3,* which in turn regulate the RAS superfamily member adenosine 5’-diphosphate ribosylation factor (ARF) [98].

## 5. Epigenetic Effect of Stilbene on Genes and Proteins Related to Cell-Proliferation and Metastasis

Unabated cell proliferation is one of the major phenotypic hallmarks of breast cancer initiation. Constitutive activation of the PI3K catalytic domain p110α due to mutations in the *PIK3CA* gene causes cell migration and metastatic properties in breast cancer cells and is a strong biomarker for the early detection of breast cancer occurrence or recurrence, and is hence used for periodic molecular mammographic screening [99,100]. PI3K is also capable of exerting epigenetic effects by controlling the histone methyltransferases like EZH2, and thereby decreasing the global methylation and genome-wide upregulation of transcription. PI3K/AKT also modulates HATs like p300/CBP and has contributions towards euchromatin−heterochromatin modulation and DNA accessibility [101,102]. Hence, the targeted inhibition of PI3K has been proven to be useful for chemoprevention and therapy in luminal breast cancer [103,104]. However, one of the notable challenges that this mutation, combined with dysregulation of other cell cycle mediators like PTEN, pose is rendering insensitivity towards traditional chemotherapeutic reagents, like lapatinib and trastuzumab, and PI3K inhibitor therapy [105,106,107]. Stilbenes such as piceatannol have effects on the AKT/mTOR pathway, similar to the EGCG and SAHA combination. There is evidence of it successfully inhibiting the PI3K pathway and significantly reducing cell proliferation and migration in prostate cancer [108], and it might be promising for formulating a PI3Ki therapy. Part of this possibility lies in the fact that two stilbenes, resveratrol and piceatannol, inhibit the JAK/STAT pathway, thereby suppressing the cell survival signals [109] and potentially reversing the chemoresistance of malignant cells to cisplatin treatment, as shown in other types of cancer [110]. Further investigation is required in order to confirm these possibilities regarding targeted breast cancer therapy and chemoprevention. There are also limited data on the epigenetic effects of stilbenes on the genes that encode the proteins of these major pathways that regulate breast cancer risk.

Ki-67 is a protein that was traditionally used as a biomarker for cell proliferation, for which high levels provide information on distinguishing women with no tumors, benign tumors, and malignant breast tumors [111]. However, recent research posits that cell cycle progression depends substantially on the localization of this protein within a mitotic cell. Sun et al. made an account of all of the past research explaining the molecular relationship between Ki-67 deficiency in a dividing cell and cell cycle arrest [112]. Dearth of Ki-67 induces cyclin-dependent kinase inhibitor checkpoint protein p21 in human primary fibroblasts and can delay S phase initiation in hTERT-BJ skin cell lines. In addition, Ki-67 plays a major role in heterochromatin organization in nucleolar periphery, thereby controlling a cell’s entry to the G1/S phase [112]. Li et al. using in vitro gastric cancer cell lines, successfully suppressed the expression of Ki-67 by targeting methylated CpG binding protein 2 (MBD2) at the methylated promoter of the Ki-67 protein-coding gene, thereby preventing the transcription factor Sp1 from binding [113]. This concept seems to hold potential for formulating a therapy or prevention of breast cancer cells. However, Sun et al. challenged this concept as it might not be applicable for all breast cancer cells. MDA-MB-231 (ER-, PR-, and HER2-) cells express a higher Ki-67 expression contrary to MCF-F7 (ER+, PR+, and HER2-) cells and have been shown to have no p21 induction [112]. Employment of stilbenes like resveratrol to repress Ki-67 transcription might also be challenging because studies show that it prevents DNMT1 and MBD2 binding at the promoter of important oncogenes like *BRCA1,* thereby activating them [114]. The potential to make resveratrol work differentially, gene-to-gene, needs further research.

## 6. Bioavailability of Stilbenes in Target Tissue and Limitations

Unlike flavones, stilbenes are derived at low or variable concentrations from edible sources. In addition, stilbenes like resveratrol have a substantially short half-life (14.4 min in mice), low water-solubility, and faster metabolization and exclusion rate, not only in the cells of test subjects, but also their gut-microbiota, which reduces the functionality of stilbene as anti-cancer compounds [29,115,116]. Depending on the chemical conjugate that the stilbenes possess in food, their bioavailability varies greatly. For example, the pharmacokinetic profile of pterostilbene is better than that of resveratrol due to the presence of methoxy groups at the 3- and 5-carbon positions of the m-hydroquinone moiety and their higher lipophilicity [29,117]. This makes the formulation of an achievable stilbene-containing diet for daily consumption a difficult task. Apart from this, extensive preclinical and clinical studies conducted on cell lines and xenografted mouse and rat models have shown that the bioavailability of stilbenes, like resveratrol, in the plasma is as low as 42.8 ± 4.4 µM after 5 min, if administered independently and intravenously at a rate of 20 mg/kg body weight [118,119]. Similar results were recorded for other stilbenes like pterostilbene, pinostilbene, and gnetol, except for the stilbene piceatannol, which has a bioavailability 2.6 times that of resveratrol. The bioavailability of stilbenes at the target tissue also varies depending on the species, route of administration (some examples include oral capsule gavage, dietary intake, and intravenous injection), age, sex, and lifestyle of the subjects [119].

By combining the results generated on human subjects by Sergides et al. [120] and Ávila-Gálvez et al. [121], it can be concluded that a daily dose between 437.7 mg to 500 mg of pure resveratrol or plant-derived resveratrol combined with other staple nutrients has been observed to effectively be utilized by the adult patient body without any cytotoxicity. Metabolite profiling [121] has revealed that the major drawback with resveratrol is that it does not reach the healthy or malignant breast tissue in its original form. The resveratrol-metabolites generated in vivo in human patients were found to be highly bioavailable in both healthy and malignant breast tissue, but were significantly unsuccessful in exerting an antiestrogenic and antiproliferative activity on both of the tissue types. These observations reduce the possibility of resveratrol and similar stilbenes that are considered as potent risk-reducing dietary chemopreventive compounds.

To address all such problems, research is ongoing to find better mechanisms to deliver stilbenes to the target tissue. It has been hypothesized that stilbenes, being hydrophobic in nature, may be transported more efficiently through lipid-based cellular delivery mechanism (liposomes) or through emulsification, thereby facilitating intestinal absorption [122]. Other methods, like combining stilbenes with sulfobutylether-β-cyclodextrin, have been shown to have increased stability and a better bioavailability of the former at the target tissue [123,124].

## 7. Conclusions

Presently, there are not enough accurate methods to detect dormant risk factors in breast tissue before the occurrence of the disease. Zubor et al. [74] suggest a multiomics approach encompassing MRI, mammography, and liquid biopsy with added emphasis on epigenetic miRNA profiling of both blood and breast tissue as the best way to address primary prevention. This makes the primary prevention of breast cancer not as popular of a concept as secondary or tertiary prevention. Besides exposing oneself to invasive medical procedures, positive outcomes upon the administration of stilbenes in relevant doses are not ascertained for every individual. There could be adverse effects that also need evaluation, especially adopting stilbene-derived synthetic chemoprevention while gestating. There has been evidence of increased possibilities of transgenerational developmental deformities [125]. Epigenetic biomarkers are subject to constant change, given the continuous change of an individual’s external and internal environment, providing a scope for drug repurposing and combinatorial therapeutic and preventive measures. Presently, there is interest in the prospects of NSAIDs, like aspirin and ibuprofen, as risk-reducing medication for breast cancer as they can inhibit the overexpression of *COX-2*, a gene responsible for the initiation of tumorigenesis and inflammation in breast epithelia [126]. Regulski et al. recently identified 19 trans-stilbene and 6 trans-4-stilbazole derivatives that can serve as a replacement for NSAIDs, showing a similar interaction at the Tyr355 residue of the COX-2 protein N-terminal and the same docking energy in simulation [127], although the detailed epigenetic mechanisms of these chemicals remain to be evaluated.

With the discovery of new biomarkers and stilbene variants, more research on novel epigenetic reactions and targets is required. It is imperative to approach the first signs of breast cancer risk with combinatorial approaches instead of monotherapy for attaining the maximum efficacy of these chemicals.

## Abbreviations

Ais	Aromatase inhibitors
Akt	Protein kinase B
ARF	Adenosine 5’-diphosphate ribosylation factor
ATM	Serine/ Threonine kinase
Bax	Bcl-2-associated X protein
Bcl-2	B-cell lymphoma 2 protein
*BRCA1/2*	Breast cancer gene 1 and 2
*CD1*	Cluster of differentiation 1
*cIAP2*	Cellular Inhibitor Of Apoptosis 2 gene
*c-Myc*	Myelocytomatosis proto-oncogene
CNA-high	Copy number alterations high
CNA-quiet	Copy number alterations quiet
*COX2*	Cyclooxygenase 2 gene
CpG	Regions of DNA with repetitive occurrence of cytosine and guanosine nucleotides
*Cyth3*	Cytohesin-3 gene
DNA	Deoxyribonucleic acid
DNMT1	DNA methyltransferase 1
DNMT3A	DNA methyltransferase 3 Alpha
DNMT3B	DNA methyltransferase 3 Beta
DNMTs	DNA methyl transferases
EGCG	Epigallocatechin gallate
EMT	Epithelial mesenchymal transformation
ER-	Estrogen receptor negative
ER+	Estrogen receptor positive
*ERBB2*	Erythroblastic leukemia viral oncogene homologue 2
ERK1/2	Extracellular signal-regulated kinases
EZH2	Enhancer of zeste homolog 2 protein
*Git2*	Guanosine triphosphatase-activating protein gene
HATs	Histone acetyltransferases
HDACi	Histone deacetylase inhibitor
HDACs	Histone deacetylases
HMTs	Histone methyltransferases
HNRNPA1	Heterogeneous nuclear ribonucleoprotein A1
*hTERT*	Human telomerase reverse transcriptase
H3R2me2a	Asymmetrical dimethylation at arginine residue 2 of histone subunit 3
HER2	Human epidermal growth factor receptor 2
H4R3me2s	Dimethylation at arginine residue 3 residue of histone subunit 4
H3ac	Acetylation at histone subunit 3
H4ac	Acetylation at histone subunit 4
HAC6	Histone deacetylase 6
H3K4me3	Trimethylation at histone subunit 3 at lysine 4
H3R8me2a	Asymmetrical dimethylation at arginine residue 8 residue of histone subunit 3
H3R8me2s	Dimethylation at arginine residue 8 residue of histone subunit 3
H4K5	Histone subunit 4 at lysine residue 5
H3K9	Histone subunit 3 at lysine residue 9
H3K9me3	Trimethylation at histone subunit 3 at lysine 9
H4K8	Histone subunit 4 at lysine residue 8
H3K9ac	Acetylation at histone subunit 3 at lysine 9
H4K12	Histone subunit 4 at lysine residue 12
H3K14	Histone subunit 3 at lysine residue 14
H4K16	Histone subunit 4 at lysine residue 16
H3K18	Histone subunit 3 at lysine residue 18
H3K23	Histone subunit 3 at lysine residue 23
H3K27me3	Trimethylation at histone subunit 3 at lysine 27
JAK	Janus kinase
Ki-67	Kiel-67 antibody
Let-7b	MicroRNA Let-7b
LINEs	Long interspersed nuclear elements
lncRNA	Long non-coding RNA
MAPK	Mitogen-activated protein kinase
MBD2	Methyl-CpG-binding domain protein 2
MeCP2	Methyl-CpG-binding protein 2
MET	Mesenchymal-to-epithelial transformation
miRNA	MicroRNA
mTOR	Mammalian target of rapamycin
N terminal	Nitrogen terminal
ncRNA	Non-coding RNA
NF-κB	Nuclear factor kappa light chain enhancer of activated B cells
NSAIDs	Non-steroidal anti-inflammatory drugs
p300/CBP	CREB-binding protein and its homolog p300
*PALB2*	Partner and localizer of BRCA2
PARP	Poly (ADP-ribose) polymerase
PEITC	Phenethyl isothiocyanate
PI3K	Phosphoinositide 3-Kinase
PI3Ki	Phosphoinositide 3-Kinase inhibitor
PIK3CA	Phosphatidylinositol-4,5-Bisphosphate 3-Kinase Catalytic Subunit Alpha
PR-	Progesterone receptor negative
PR+	Progesterone receptor positive
*PTEN*	Phosphatase and Tensin homolog deleted on chromosome 10
RAS	Rat sarcoma
RNA	Ribonucleic acid
RNAi	Interfering RNA
SAHA	Suberanilohydroxamic acid
*SALL3*	Spalt Like Transcription Factor 3 coding gene
*SEMA3A*	Semaphorin 3A gene
SERMs	Selective estrogen receptor modulators
siRNA	Small interfering RNA
SIRT1	Sirtuin 1
SNPs	Single nucleotide polymorphism
STAT	Signal transducer and activator of transcription proteins
*STK11*	Serine/threonine kinase 11
TNBC	Triple-negative breast cancer
*TP53*	Tumor protein 53
TSGs	Tumor suppressor genes
Tyr355	Tyrosine 355 residue
